# Ultrasonic optic disc height combined with the optic nerve sheath diameter as a promising non-invasive marker of elevated intracranial pressure

**DOI:** 10.3389/fphys.2023.957758

**Published:** 2023-03-10

**Authors:** Ze-yang Yu, Ying-qi Xing, Cong Li, Si-bo Wang, Xiao-nan Song, Cui-cui Wang, Li-juan Wang

**Affiliations:** ^1^ Department of Neurology, The First Hospiatal of Jilin University, Changchun, China; ^2^ Department of Rehabilitation Medicine, Xiangyang Central Hospital, Affiliated Hospital of Hubei University of Arts and Science, Xiangyang, China; ^3^ Department of Vascular Ultrasonography, Xuanwu Hospital, Capital Medical University, Bejing, China

**Keywords:** intracranial hypertension, optic nerve sheath diameter, optic disc height, intracranial pressure, ultrasound

## Abstract

**Background/aim:** Patients with elevated intracranial pressure (ICP) tend to have optic disc edema and a thicker optic nerve sheath diameter (ONSD). However, the cut-off value of the optic disc height (ODH) for evaluating elevated ICP is not clear. This study was conducted to evaluate ultrasonic ODH and to investigate the reliability of ODH and ONSD for elevated ICP.

**Methods:** Patients suspected of having increased ICP and who underwent a lumbar puncture were recruited. ODH and ONSD were measured before lumbar puncture. Patients were divided according to elevated and normal ICP. We analyzed the correlations between ODH, ONSD, and ICP. ODH and ONSD cut-off points for the identification of elevated ICP were determined and compared.

**Results:** There were a total of 107 patients recruited for this study, 55 patients with elevated ICP and 52 with normal ICP. Both ODH and ONSD in the elevated ICP group were higher than in the normal group [ODH: median 0.81 (range 0.60–1.06) mm vs. 0.40 [0–0.60] mm, *p* < 0.001; ONSD: 5.01 ± 0.37 mm vs. 4.20 ± 0.38 mm, *p* < 0.001]. ICP was positively correlated with ODH (*r* = 0.613; *p* < 0.001) and ONSD (*r* = 0.792; *p* < 0.001). The cut-off values of ODH and ONSD for evaluating elevated ICP were 0.63 mm and 4.68 mm, respectively, with 73% and 84% sensitivity and 83% and 94% specificity, respectively. ODH combined with ONSD showed the highest value under the receiver operating characteristic curve of 0.965 with a sensitivity of 93% and a specificity of 92%.

**Conclusion:** Ultrasonic ODH combined with ONSD may help monitor elevated ICP non-invasively.

## 1 Introduction

Elevated intracranial pressure (ICP) is a common emergency condition associated with poor clinical outcomes or even death (Freeman, 2015). Currently, invasive ICP monitoring is still the “criterion standard” for measuring ICP. However, it is not routinely undertaken due to the lack of neurosurgeons and contraindications, such as coagulopathy or thrombocythemia. Additionally, complications such as bleeding (7%) (Bauer et al., 2011) and infection (5%–20%) (Beer et al., 2008) might reduce the feasibility of invasive ICP monitoring. Thus, a non-invasive, reproducible, and simple bedside technique for the evaluation of increased ICP is urgently needed. In recent years, ocular ultrasonography has been used to assess the increased ICP.

Optic disc edema is a common clinical manifestation of elevated ICP due to increasing cerebrospinal fluid pressure in the optic nerve sheath and swelling of nerve fibers (Hayreh, 2016). In clinical work, a high ICP value is often assessed by examining papilledema. Spectral domain optical coherence tomography (OCT) is a common method for evaluating the papillary bulge. Optic disc analysis can be widely used in the diagnosis of intracranial hypertension, but OCT images need to be evaluated by an expert examiner (Rosa et al., 2022a). Ultrasound equipment is more common and can be used at the point of care. Ultrasound is able to detect optic discs with hyperechoic lesions, which are highly reflective structures in the head of the optic nerve (Bakola et al., 2021). A few studies have reported that ultrasound could detect elevated optic discs (Lochner et al., 2019). The optic nerve sheath is derived from the three layers of the meninges, and the pressure change in the intraorbital subarachnoid space is the same as that of the intracranial subarachnoid space. When ICP increases, the optic nerve sheath expands and thickens. Many studies have confirmed that the ultrasound measurement of the optic nerve sheath diameter (ONSD) is a non-invasive, dynamic, and instantaneous bedside measurement to assess ICP (Wang et al., 2018; Chen et al., 2019). However, the criteria for ultrasonographic measures of the ONSD to assess increased ICP have not reached a consensus. Several studies have reported a cut-off point of 5 mm for the identification of increased ICP or elevated opening pressure on the lumbar puncture (Blaivas et al., 2003; Kim et al., 2021), while some studies have reported a cut-off of less than 5 mm (Maude et al., 2013). The purpose of this study is to first identify the optimal cut-off values for ODH and ONSD to evaluate ICP, and the second is to investigate whether the combination of the two can improve the effectiveness of non-invasive evaluation of intracranial hypertension.

## 2 Methods

### 2.1 Study population

This study recruited patients who were suspected of having increased ICP for various reasons and underwent lumbar puncture from April 2019 to November 2020 in the Department of Neurology of the First Hospital of Jilin University. The study has been approved by the Ethics Committee of the First Hospital of Jilin University (No.19K128-001). All patients voluntarily participated and signed an informed consent form by themselves or their families. The exclusion criteria were as follows: 1) <16 years old; 2) eye diseases such as glaucoma, eye trauma, and tumor; 3) the patient was convulsing or restless and could not cooperate with the examination; 4) patients with lumbar puncture contraindications, such as posterior fossa mass lesion, lumbar epidural abscess or subdural empyema, spinal subarachnoid block, and platelet counts of less than 50,000 (Roos, 2003). We recorded sex, age, body mass index (BMI), systolic blood pressure (SBP), diastolic blood pressure (DBP), mean arterial blood pressure (MABP), waist circumference, head circumference, ICP, ONSD, ODH, etiology, disease duration, and the presence or absence of sedation, and mechanical ventilation in all patients.

### 2.2 Measurements

#### 2.2.1 Ultrasonography

**FIGURE 1:**
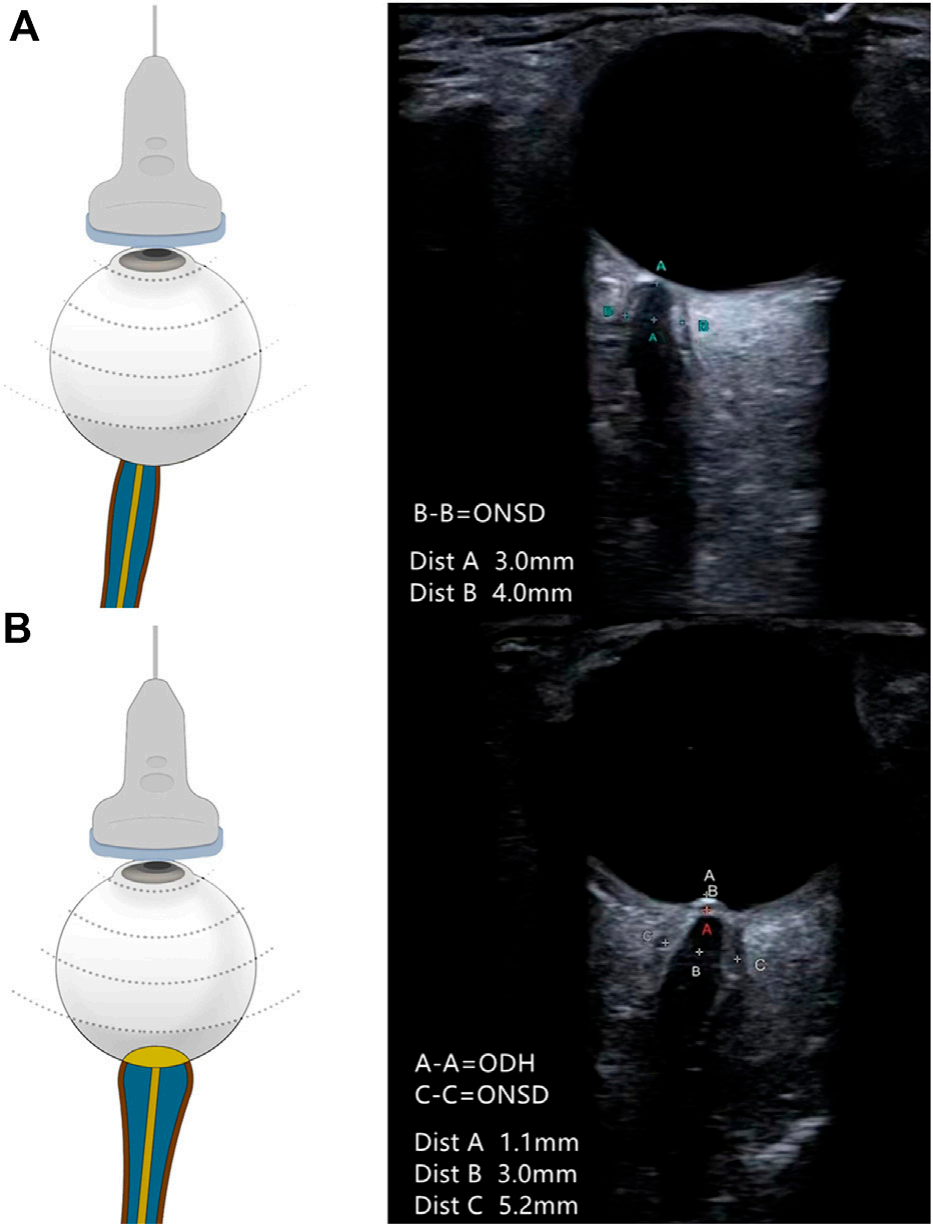
Schematic representation of the ultrasound placement and the representative ultrasound image. **(A)** ONSD measured in a patient with normal ICP. ONSD measured at 3-mm retro-orbital (A-A) was 4.0 mm (B-B). **(B)** ONSD and ODH measured in a patient with elevated ICP. ONSD measured at 3-mm retro-orbital (B-B) was 5.2 mm (C-C). ODH was 1.1 mm (A-A). The cross at point A in red is the location of the “strongly reflecting line.”

Ultrasonography was performed 10 min before the lumbar puncture. All patients underwent measurement with a multifunctional vascular ultrasound system (Aplio 500 TUS-A500; Canon Medical Systems, Otawara, Japan), using a 5–14-MHz probe in the B-mode. The output power of the machine was adjusted according to the ALARA (“as low as reasonably achievable”) principle (Toms, 2006). The patient was instructed to lie in the supine position, lightly close their eyes, and to keep their eyes as still as possible. Then, the observer held the probe and gently placed it on the patient’s closed eyelids to avoid excessive pressure on the eyeballs. All participants were examined by two experienced observers (ZY and LW) who were blind to the ultrasonographic results. ODH was measured between the fundus and the dome of the optic disc (from the uppermost part of the swollen disc to the strongly reflecting line which represents the lamina cribrosa) (Lochner et al., 2016). ONSD was measured from 3 mm behind the eyeball and perpendicular to the optic nerve axis (Figure 1). (Geeraerts et al., 2008) The measurement was performed in both the transverse and vertical sections. Eight measurement values were obtained for both eyes. To reduce measurement errors, the final ONSD and ODH values were averaged.

#### 2.2.2 Lumbar puncture

Lumbar puncture was performed within 10 min after the ultrasound examination. Lumbar puncture was completed by an experienced neurologist in accordance with our hospital’s standard procedure. The patients were placed in the left lateral decubitus position with the neck and knees flexed. L3-L4 or L4-L5 was selected as puncture points, and after disinfection, drape, and local anesthesia, a 9-G puncture needle was used for puncture along the anesthesia hole. After seeing the outflow of the cerebrospinal fluid, connecting the pressure-measuring tube, and making the patient straighten their legs slowly, the ICP was read after the water column stabilized. Patients were divided into elevated and normal ICP groups, where ICP >200 mm H_2_O was considered to be increased (Kimberly et al., 2008).

### 2.3 Statistical analyses

The Kolmogorov–Smirnov test was used to test for the normality of continuous variables. Continuous variables with normal distribution are represented as means ± standard deviations (SDs). Continuous variables with non-normal distribution are represented by medians and interquartile ranges (IQRs). Categorical variables are presented as frequencies (percentages). Normally distributed continuous variables were compared between groups using Student’s t-test and non-normally distributed variables using the Wilcoxon rank-sum test. The chi-squared test was used to compare categorical variables among the groups. Bland–Altman analysis was used to analyze the reliability among the observers. The relationships between ICP and ODH and between ICP and ONSD were evaluated using Spearman correlation analysis. A multiple linear regression model was established to determine independent parameters that affect ICP. Receiver operating characteristic (ROC) curves were established to obtain the area under the curve (AUROC), the best cut-off value, and the corresponding sensitivity and specificity. DeLong’s test was used to analyze the differences between the ROC curves. All statistical analyses were conducted using SPSS version 22.0 (IBM, Armonk, NY) and MedCalc (version 15.2.2; MedCalc, Mariakerke, Belgium). Statistical significance was set at *p* < 0.05.

## 3 Results

The study included a total of 107 patients aged 16–80 years (44.96 ± 17.14 years) and 62 males (58%). The suspected reasons for having increased ICP included intracranial infection (*n* = 77), venous sinus thrombosis (*n* = 13), peripheral neuropathy (*n* = 7), and cerebrovascular disease (*n* = 10). The disease duration ranged from 1 to 65 days, with a median of 11 days. ICP ranged from 100 to 400 mm H_2_O: the mean ICP in the normal and elevated groups was 149.23 ± 22.43 mm H_2_O and 294.91 ± 65.29 mm H_2_O, respectively. There were 52 patients in the normal ICP group and 55 in the elevated ICP group. Neither sex, age, BMI, waist circumference, head circumference, SBP, DBP, MABP, and sedation nor mechanical ventilation was significantly different among groups (Table 1).

**TABLE 1:** Demographic and baseline characteristics.

	Normal (n = 52)	Elevated (n = 55)	*p*-value
Male, n (%)	26 (50%)	36 (65.5%)	0.106
Age (years), mean (SD)	48.2 (17.1)	41.9 (16.8)	0.055
BMI (kg/m^2^), mean (SD)	22.86 (3.10)	24.03 (3.25)	0.059
Waist circumference (cm), mean (SD)	79.79 (9.34)	82.84 (10.02)	0.107
Head circumference (cm)	57 (56–58)	57 (56–58)	0.522
Median (IQR)			
SBP (mmHg), mean (SD)	126.04 (20.00)	131.73 (16.345)	0.109
DBP (mmHg), mean (SD)	74.06 (13.52)	77.78 (14.09)	0.166
MABP(mmHg), mean (SD)	91.38 (12.75)	95.76 (13.62)	0.089
Sedation, n (%)	9 (17.3%)	8 (14.5%)	0.696
Mechanical ventilation, n (%)	4 (7.7%)	8 (14.5%)	0.414
ODH (mm), median (IQR)	0.40 (0–0.60)	0.81 (0.60–1.06)	<0.001
ONSD (mm), mean (SD)	4.20 (0.38)	5.01 (0.37)	<0.001

SD, standard deviation; IQR, interquartile range; BMI, body mass index; SBP, systolic blood pressure; DBP, diastolic blood pressure; MABP, mean arterial blood pressure; ODH, optic disc height; ONSD, optic nerve sheath diameter.

All patients completed ODH and ONSD measurements. The Spearman correlation coefficient for ODH between the two observers was 0.921. The average ODH difference between the two observers by Bland–Altman analysis was −0.008 (0.148) mm. The limits of agreement were 0.282 mm in the left eye and −0.298 mm in the right eye. There was no statistically significant difference in the ONSD between the left and right eyes in all patients (*p* = 0.89). The Pearson correlation coefficients of the ONSD measured between the left and right eyes of the two observers were 0.841 and 0.840, respectively. The average ONSD difference between the two observers by Bland–Altman analysis was −0.012 (0.369) mm. The limits of agreement were 0.711 mm in the left eye and −0.735 mm in the right eye.

The ODH range of all patients was 0–1.90 mm. The median ODH in the elevated ICP group was 0.81 (0.60–1.06) mm, which was higher than that in the normal ICP group (0.40 [0–0.60] mm, *p* < 0.001). There was a positive correlation between ICP and ODH (*r* = 0.613; *p* < 0.001). The ONSD range of all patients was 3.47–5.87 mm; the average ONSD of the elevated ICP group was 5.01 ± 0.37 mm, which was significantly higher than that of the normal ICP group (4.20 ± 0.38 mm, *p* < 0.001). There was a positive correlation between ICP and the ONSD (*r* = 0.792, *p* < 0.001).

For multiple linear regressions, ICP was used as the dependent variable and factors with *p* < 0.1, including age, BMI, head circumference, MABP, ODH, and ONSD were selected as independent variables. Finally, only the ODH and ONSD remained in the model, yielding the following regression equation: ICP = 95.459×ONSD+68.066×ODH-259.451. The analysis of variance showed that the aforementioned model was significantly different (F = 97.331; *p* < 0.001). The adjusted *R* ^2^ value was 0.645, and the Durbin–Watson value was 1.972. The residuals conformed to a normal distribution.

**FIGURE 2:**
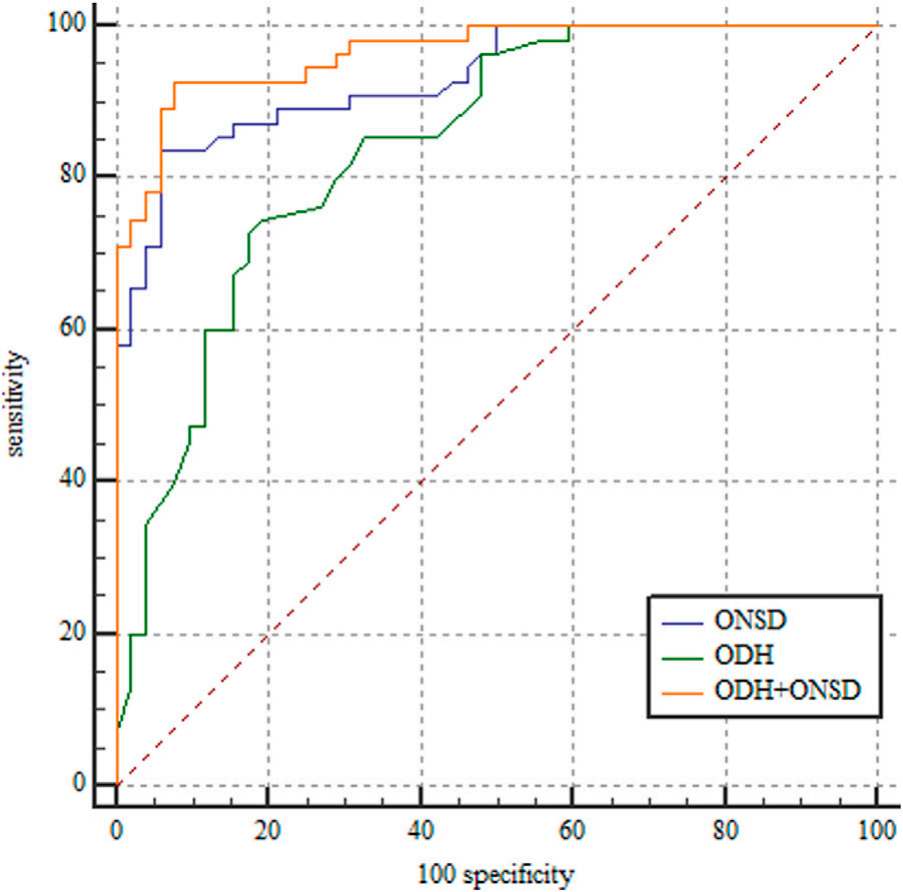
Receiver operating characteristic curves for ODH, ONSD, and ODH + ONSD. ODH: AUROC, 0.842 (95% CI: 0.768–0.916). ONSD: AUROC, 0.932 (95% CI: 0.887–0.977). ODH + ONSD: AUROC, 0.965 (95% CI: 0.936–0.994). ONSD, optic nerve sheath diameter; ODH, optic disc height; AUROC, area under the receiver operating characteristic; CI, confidence interval.

ROC curves were generated based on a standard ICP >200 mm H_2_O. We obtained ROC curves for the three models (Figure 2), and all three models could be used to diagnose elevated ICP. The best cut-off value for the ODH was 0.63 mm, with 73% sensitivity and 83% specificity. The best cut-off value for the ONSD was 4.68 mm, with 84% sensitivity and 94% specificity. The sensitivity and specificity of the ODH combined with the ONSD in the diagnosis of elevated ICP were 93% and 92%, respectively. The AUROCs of the ODH alone, ONSD alone, and the ODH combined with the ONSD were 0.842 (95% confidence interval [CI]: 0.768–0.916), 0.932 (95% CI: 0.887–0.977), and 0.965 (95% CI: 0.936–0.994), respectively. For evaluating elevated ICP, the ONSD was superior to the ODH (*p* = 0.039) in evaluating elevated ICP, and the ODH combined with the ONSD was superior to both the ODH (*p* < 0.001) and ONSD (*p* = 0.031).

## 4 Discussion

Both the ODH and ONSD measured by ultrasound can be used to evaluate elevated ICP, and there is a positive correlation between the ODH and ICP and between the ONSD and ICP. Although the ODH is not as sensitive as the ONSD, the combined evaluation of the two can improve the sensitivity of detection of elevated ICP.

Few studies have used ultrasound to measure the ODH to evaluate elevated ICP. In a report of four patients with elevated ICP, emergency ultrasound found thickening of the ONSD and the presence of the optic disc, suggesting the appearance of the optic disc in patients with elevated ICP is helpful for the evaluation of ICP (Marchese et al., 2015). This case series showed that an elevated optic disc is a new and useful discovery in the evaluation of elevated ICP. Ebraheim et al. (2018) conducted a study on 54 patients and found a positive correlation between ICP and the ODH (*r* = 0.572; *p* = 0.004). We also obtained a correlation between ICP and the ODH in 107 patients (*r* = 0.613; *p* < 0.001). A previous study found an elevated optic disc when using ultrasound to measure the ONSD to evaluate ICP and used the presence or absence of the optic disc as the basis for the diagnosis of elevated ICP. Although the specificity of the diagnosis was 100%, the sensitivity was only 41.6% (Mohson and Auday, 2019). In our study, ODH was specifically measured, and the sensitivity and specificity were 73% and 83%, respectively. Therefore, the ODH was better than assessing the presence of the optic disc for evaluating elevated ICP. In addition, few studies have confirmed the cut-off value of the ODH for elevated ICP. Teismann et al. (2013) studied the ability of the ultrasound measurement of the ODH to assess optic disc edema in 14 samples. The identified cut-off value was 0.6 mm for optic disc edema, and the sensitivity and specificity were 82% and 76%, respectively. Our study identified a cut-off value of 0.63 mm for elevated ICP, which is similar to that recorded by Teismann et al. Teismann et al. used ophthalmoscopy as the diagnostic criterion, and our study used lumbar puncture pressure as the criterion.

Lumbar puncture is a relatively common invasive procedure, but there are certain contraindications. If the patient has a local infection or thrombocytopenia, lumbar puncture cannot be performed. With the development of ultrasound technology, measuring the ONSD can be used as a non-invasive way to observe ICP in many diseases, such as tuberculous meningitis (Sangani and Parikh, 2015), idiopathic high ICP (Marchese et al., 2015), cerebral hemorrhage (Naldi et al., 2019), and spontaneous low ICP (Fichtner et al., 2019). The ONSD is useful for the evaluation of elevated ICP. Geeraerts et al. (2008) found a significant correlation between ICP and the ONSD through 78 measurements (*r* = 0.71; *p* < 0.0001). Our study also confirmed a good correlation between the ONSD and ICP in 107 patients (*r* = 0.792; *p* < 0.001). In this study, 4.68 mm was used as the cut-off value, which is lower than that previously reported. This may be due to ethnic differences. A study from Bangladesh proposed 4.75 mm as a cut-off value for diagnosing elevated ICP (Maude et al., 2013). Bangladesh and China are also Asian countries, so the ethnicity of the studied population is Asian and the cut-off values -obtained are similar. More data may be needed to establish different cut-off value standards among different ethnicities.

Most previous studies using ocular ultrasound to evaluate elevated ICP focused on the study of the ONSD; only the optic disc was described, its efficacy was not tested, and the two methods were not compared or combined. To solve these problems, we carried out comparison and joint analyses of the ODH and ONSD. We found that the ONSD is superior to the ODH in evaluating elevated ICP in terms of sensitivity and specificity. In addition, our study found that the sensitivity of the ODH combined with the ONSD in evaluating elevated ICP was higher than either method alone. The sensitivities of ODH and ONSD were 73% and 84%, respectively, and the combined sensitivity of the two was 93%.

Compared with other non-invasive methods for evaluating elevated ICP, ultrasound has certain advantages. It is an examination method that can be quickly learned, quantified, and performed at the bedside. Although an ophthalmoscope can be used to directly observe the optic disc at the bedside, it cannot make a quantitative measurement. In critically ill patients who cannot undergo invasive ICP measurements or are not suitable for transport, non-invasive measurements of the ONSD and ODH have a significant advantage in assessing ICP. Ultrasound measurement of the ONSD is easy to learn and can be taught to novices quickly and easily. After just a 4-h lecture, a novice’s measurement result will be similar to that of an expert’s in a week (Potgieter et al., 2011). Junior sonographers can accurately measure the ONSD within a short training period (Betcher et al., 2018). Ultrasound combines the aforementioned advantages, which can conveniently measure the ODH and ONSD at the bedside. OCT can also be used to assess optic disc drusen, and a study has compared the two. Optic nerve ultrasound remains the most reliable method (Rosa et al., 2022a).

In recent years, some studies have used the ONSD and other image markers to improve the diagnostic accuracy of high intracranial pressure and its associated diseases in both adults and children. The ratio of the ONSD to eyeball transverse diameter (ETD) by ultrasound may be a reliable predictor of intracranial hypertension in patients with TBI (Du et al., 2020). In addition, the ONSD/ETD ratio may be more valuable than the ONSD in predicting malignant progression in patients with acute stroke (Guo et al., 2022). These methods have been studied not only in adults but also in children. Ultrasound ONSD measurements and ONSD/ETD ratios are also very sensitive to elevated ICP in children with head injury (Şık et al., 2022). A growing body of research has begun to identify more markers that may assist in the assessment of high intracranial pressure and thus improve the accuracy of the assessment of high intracranial pressure. In addition to measuring the ONSD and identifying the optic disc bulge, ocular ultrasound has indicated a further marker, the crescent sign, which is less sensitive but has high specificity (100%) (Mohson and Auday, 2019). A recent study showed that the deformability index (DI) is a dynamic parameter that quantifies the pulsatile nature of optic nerve sheaths and can distinguish between patients with elevated and normal ICP. Joint analysis of DI and ONSD can improve a correlation with ICP (Padayachy et al., 2019).

Our study had certain limitations. First of all, the patients we enrolled in the group need to obtain the intracranial pressure value through lumbar puncture; we, therefore, excluded patients with contraindications to lumbar puncture or those who refused lumbar puncture. The relationship between the optic nerve sheath and papilla of these patients and intracranial pressure is not clear. Another limitation to this study is that most patients underwent only a single lumbar puncture. It may be better if continuous ICP monitoring can be performed, and ultrasound parameters can be measured repeatedly and dynamically. This allows us to observe whether the ONSD and ODH change dynamically with ICP or if they can represent changes in ICP over time. A better understanding and discovery of ICP change at different periods of disease. The “blooming effect” in B-scan needs to be treated with caution. A study has pointed out that when evaluating the optic nerve sheath, the standardized A-scan has some advantages. (Rosa et al., 2022b). Moreover, more in-depth research is needed.

## 5 Conclusion

This preliminary study showed that ultrasound measurement of the ODH and ONSD is a non-invasive bedside measurement for the rapid evaluation of elevated ICP, and the combination of the two can be used as a new marker for evaluating elevated ICP.

## Data Availability

The raw data supporting the conclusion of this article will be made available by the authors, without undue reservation.
